# Impact of Interleukin‐1 Blockade on the Development of Macrophage Activation Syndrome in Still Disease: Incidence and Diagnostic Validity of the EULAR/ACR/PRINTO 2016 MAS Classification Criteria

**DOI:** 10.1002/art.43263

**Published:** 2025-08-09

**Authors:** Remco G. A. Erkens, Greta Rogani, Laura Huber, Anouk Verwoerd, Dieneke Schonenberg‐Meinema, J. Merlijn van den Berg, Wineke Armbrust, G. Elizabeth Legger, Sylvia Kamphuis, Ellen J. H. Schatorjé, Esther P. A. H. Hoppenreijs, Joost F. Swart, Marc H. A. Jansen, Jorg van Loosdregt, Sebastiaan J. Vastert

**Affiliations:** ^1^ Division of Pediatric Rheumatology and Immunology, Wilhelmina Children's Hospital and Center for Translational Immunology University Medical Center Utrecht, Utrecht University Utrecht The Netherlands; ^2^ Division of Pediatric Rheumatology and Immunology, Wilhelmina Children's Hospital University Medical Center Utrecht, Utrecht University Utrecht The Netherlands; ^3^ Division of Pediatric Immunology, Rheumatology and Infectious Diseases, Emma Children's Hospital, Amsterdam University Medical Centers University of Amsterdam Amsterdam The Netherlands; ^4^ Division of Pediatric Rheumatology and Immunology, Beatrix Children's Hospital University Medical Center Groningen Groningen The Netherlands; ^5^ Division of Pediatric Rheumatology, Sophia Children's Hospital Erasmus Medical Center Rotterdam The Netherlands; ^6^ Department of Pediatric Rheumatology, Amalia Children's Hospital Radboud University Medical Center Nijmegen The Netherlands; ^7^ Center for Translational Immunology University Medical Center Utrecht, Utrecht University Utrecht The Netherlands

## Abstract

**Objective:**

To evaluate the applicability of the 2016 European Alliance of Associations for Rheumatology (EULAR)/American College of Rheumatology (ACR)/Paediatric Rheumatology International Trials Organisation (PRINTO) macrophage activation syndrome (MAS) classification criteria in patients with Still disease and systemic juvenile idiopathic arthritis (sJIA‐SD) treated with interleukin‐1 (IL‐1)–targeted therapy and to assess the incidence of MAS in this context.

**Methods:**

We analyzed retrospective and prospective data from Dutch patients with sJIA‐SD (diagnosis 2008–2017, n = 54) and data from a nationwide prospective Dutch cohort and intervention study (diagnosis 2017–2022, n = 66). From these cohorts, MAS episodes developing in patients with sJIA‐SD treated with IL‐1–targeted therapy (anakinra or canakinumab) with at least two years of follow‐up were selected. Clinical and laboratory data were extracted from the electronic patient files.

**Results:**

A total of 22 patients experienced 29 MAS episodes while receiving IL‐1–targeted treatment. Seven patients had recurrent MAS episodes (not all while receiving IL‐1 blockade). The 2016 criteria for MAS in sJIA‐SD were met for 28 of 29 MAS episodes (97%). Within the prospective nationwide cohort starting anakinra as first‐line monotherapy, the incidence rate of MAS in the first two years of disease was 18% (12 of 66 patients, with 11 of 12 while receiving IL‐1 inhibition). This incidence is comparable to that observed in historical glucocorticoid‐treated patients. Half of MAS episodes occurred within three months after diagnosis, and Epstein‐Barr virus was the most common identifiable trigger.

**Conclusion:**

Although first‐line anakinra in new‐onset sJIA‐SD has demonstrated high response rates, our data suggest the incidence of MAS in the first two years of disease is not reduced. Patients appear to be particularly at risk early in disease. Importantly, our data show that the EULAR/ACR/PRINTO 2016 MAS classification criteria remain applicable to patients receiving IL‐1–targeted therapy.

## INTRODUCTION

Still disease (SD), which includes systemic juvenile idiopathic arthritis (sJIA) in children and adult‐onset SD in adults,^1^ is a severe disorder with autoinflammatory characteristics.^2^, ^3^, ^4^ Over the past two decades, biologic therapies targeting key inflammatory pathways have significantly improved outcomes for affected children while reducing dependence on glucocorticoids.^5^, ^6^, ^7^, ^8^, ^9^, ^10^ Among these targeted treatments, notable agents include anakinra, a recombinant interleukin‐1 (IL‐1) receptor antagonist; canakinumab, a monoclonal antibody targeting IL‐1β; and tocilizumab, a monoclonal antibody against the IL‐6 receptor.

Since 2008, anakinra has been used as a first‐line treatment for sJIA‐SD in the Netherlands, demonstrating high efficacy.^6^, ^11^ Nearly 90% of patients achieve clinically inactive disease at one point in time after 12 months (70% with anakinra monotherapy), and ~50% of patients are able to taper off anakinra therapy within the first year while maintaining long‐term disease remission.^6^, ^11^, ^12^ However, despite these advancements, approximately 25% to 30% of patients with sJIA‐SD ultimately develop a (variable and) more refractory disease course, necessitating (combined) maintenance treatment.^12^

One of the most notable complications of SD is macrophage activation syndrome (MAS), a form of secondary hemophagocytic lymphohistiocytosis (HLH) in the context of rheumatic disease.^13^, ^14^, ^15^ MAS is characterized by a state of hyperinflammation marked by elevated levels of IL‐18 and interferon‐γ (IFNγ), leading to uncontrolled activation of macrophages and T lymphocytes.^14^, ^15^, ^16^ Although MAS often arises as a complication of (active) SD without a clear trigger, infections, most often viral, or treatment modifications have been associated with MAS development.^14^, ^17^ Overt MAS occurs in 10% to 20% of patients with SD, whereas 30% to 40% may display (subclinical) MAS features accompanied by specific cytokine profiles.^17^, ^18^, ^19^, ^20^, ^21^ Additionally, patients experiencing multiple MAS episodes appear to be at increased risk of developing other life‐threatening complications, such as interstitial lung disease (sJIA‐associated lung disease [sJIA‐LD]).^13^, ^22^

Although the use of targeted biologics has been proven effective in treating SD itself, it is unclear so far whether these treatments are able to significantly decrease the incidence of SD‐associated MAS. Grom et al^18^ reported that canakinumab did not significantly affect MAS risk in clinical trial settings mainly involving patients with sJIA‐SD at a later stage of their disease. To date, the incidence of MAS in patients receiving first‐line anakinra has not been reported, probably because of a lack of large prospectively observed inception cohorts with sJIA‐SD. The Dutch national prospective cohort study ESTIS (Early Stop of Targeted Treatment in Children with Systemic Juvenile Idiopathic Arthritis), following the national treatment protocol for sJIA‐SD (developed in 2017), provides a unique opportunity to investigate MAS incidence in patients initiating first‐line monotherapy with targeted biologic agents early in the disease course.

Given the significant mortality rate (up to 10%) of MAS, early diagnosis and prompt treatment, including the start of high‐dose glucocorticoids in combination with targeted treatments, is critical.^15^, ^17^, ^23^, ^24^ However, diagnosing MAS is often challenging due to overlapping clinical and laboratory features with active SD or sepsis.^25^ To facilitate early MAS identification in sJIA‐SD, the European Alliance of Associations for Rheumatology (EULAR)/American College of Rheumatology (ACR)/Paediatric Rheumatology International Trials Organisation (PRINTO) developed the 2016 MAS classification criteria.^26^ These criteria include fever, ferritin levels >684 ng/mL, and at least two of the following: platelet count ≤181 × 10^9^/L, aspartate aminotransferase (AST) levels >48 U/L, triglyceride levels >156 mg/dL, and/or fibrinogen levels ≤360 mg/dL. These criteria were initially validated in patients predominantly treated with glucocorticoids (often combined with conventional disease‐modifying antirheumatic drugs [DMARDs]), so only limited data are available on their applicability in patients with sJIA‐SD undergoing biologic treatment.

Recent studies suggest that biologic therapies may influence clinical and laboratory features of MAS. For example, patients treated with tocilizumab were less likely to experience fever and had lower C‐reactive protein (CRP) and ferritin levels, potentially impacting the applicability of the EULAR/ACR/PRINTO 2016 criteria in this population.^27^, ^28^ Schulert et al^27^ found that 43.3% of patients with MAS treated with tocilizumab did not meet the classification criteria, raising concerns about their clinical utility in the biologic treatment context. How these criteria perform in patients with sJIA‐SD treated with IL‐1–targeting biologics remains unclear, necessitating further investigation.

In this multicenter descriptive cohort study, we aim to characterize the clinical and laboratory features of patients with sJIA‐SD who developed MAS while receiving IL‐1 pathway–blocking treatment (either anakinra or canakinumab). We analyzed whether these episodes fulfilled the EULAR/ACR/PRINTO criteria for sJIA‐SD–associated MAS. Additionally, we assessed the impact of first‐line anakinra treatment on MAS incidence within the first two years of disease. Finally, we describe treatment strategies and outcomes for these patients and identify potential risk factors for MAS development. Collectively, these data will provide novel insights into features and outcomes of MAS in patients treated with IL‐1–targeting therapies and further assess the relevance of the EULAR/ACR/PRINTO MAS 2016 criteria in this population.

## METHODS

This multicenter descriptive cohort study examined patients with sJIA‐SD developing MAS while receiving IL‐1–blocking treatment (anakinra or canakinumab) from the following cohorts: The “Utrecht cohort” includes prospectively observed patients with sJIA‐SD who presented to the University Medical Center Utrecht (UMCU) with a new diagnosis of sJIA from 2008 to 2022 and started first‐line anakinra monotherapy under a standardized protocol.^6^, ^11^ In short, patients were initiated on anakinra at 2 mg/kg/day subcutaneously (on‐label dose, maximum 100 mg/day for patients ≥50 kg). If fever persisted after three days, the dose was increased to 4 mg/kg/day (maximum 200 mg/day). For patients with ongoing disease activity despite anakinra monotherapy, prednisolone (0.5–1 mg/kg/day) was added, or treatment was switched to an alternative biologic agent: canakinumab (4 mg/kg) or tocilizumab (8 mg/kg for patients >30 kg, 12 mg/kg for patients <30 kg). This treatment strategy aligns with Dutch national standards of care. The ESTIS (EudraCT identifier 2015‐004393‐16) cohort, a nationwide prospective multicenter cohort and intervention study, enrolled therapy‐naive (nonsteroidal anti‐inflammatory drugs allowed) patients newly diagnosed with sJIA‐SD starting first‐line anakinra monotherapy (2017–2022) from all six academic pediatric rheumatology centers in the Netherlands: Amsterdam University Medical Center (AUMC), Radboud University Medical Center (Radboudumc; Nijmegen), Erasmus University Medical Center (Erasmus MC; Rotterdam), University Medical Center Groningen (UMCG; Groningen), Leiden University Medical Center (LUMC; Leiden), and UMCU (Utrecht). ESTIS patients in follow‐up by the UMCU who were also included in the Utrecht cohort were counted only once. Additionally, we retrospectively identified patients who developed MAS during IL‐1–targeted treatment (diagnosis 2008–2017) from AUMC, Radboudumc, Erasmus MC, UMCG, and LUMC. All patients met the PRINTO criteria for sJIA^29^ and had a minimum follow‐up of two years. We collected demographic, clinical, and laboratory data (including regular measurements of leucocytes, differentiation counts, CRP, erythrocyte sedimentation rate [ESR], ferritin, and IL‐18) on MAS episodes from electronic medical records of all patients.

The MAS diagnosis was made clinically at the discretion of the treating pediatric rheumatology teams and always led to the initiation of systemic glucocorticoids as part of the treatment regimen. All patients underwent infectious screening at MAS onset, including blood cultures and serologic and polymerase chain reaction (PCR) testing for Epstein‐Barr virus (EBV), cytomegalovirus, and, in most cases, parvovirus B19. Genetic screening for primary immune deficiencies, including primary HLH‐related genes, was performed in all but three patients (patients 5, 6, and 7). All cytokines (IL‐18, galectin‐9 and CXCL10) were measured routinely in the UMCU clinical diagnostic laboratory, using standard operating protocols for taking, processing, and shipping samples.

The time of diagnosis of sJIA‐SD was defined as the start date of anakinra treatment, whereas time of MAS diagnosis was defined as the initiation date of glucocorticoids for the indication MAS. The follow‐up duration was defined as the time from diagnosis (anakinra initiation) to the last recorded visit with laboratory measurements before December 2024. Overall, MAS episode outcomes were assessed until the last recorded visit before December 2024 or until the occurrence of a new MAS episode (in patients with recurrent MAS episodes). For the description of the prospective ESTIS cohort, we analyzed data from the first visit (start of first‐line anakinra) to the final two‐year follow‐up visit.

The study adhered to the Declaration of Helsinki guidelines (World Medical Association, Ethics Unit, Declaration of Helsinki 2024; www.wma.net/policies‐post/wma‐declaration‐of‐helsinki/). Written informed consent was obtained from all patients and/or their legal representatives. Institutional review board approval was granted by the UMCU ethics committee (study protocols 08/215, 11/499, and 16/178).

Continuous variables are presented as medians with interquartile ranges (IQRs). Differences between two groups for nonnormally distributed data were analyzed using the Wilcoxon test and the Mann‐Whitney U test. Categorical variable differences were assessed using Pearson's chi‐square test or Fisher's exact test, as appropriate. Statistical analyses and graphical representations were performed using RStudio Version 2022.12.0+353 and R version 4.2.2 (2022‐10‐31). A *P* value below 0.05 was considered statistically significant.

## RESULTS

### Cohort characteristics

The Utrecht cohort included 50 patients newly diagnosed with sJIA‐SD, of whom 9 developed MAS. The prospective ESTIS cohort comprised 66 patients with new‐onset sJIA‐SD from six pediatric rheumatology centers in the Netherlands, 12 of whom developed MAS. Additionally, four patients who developed MAS while receiving IL‐1 pathway–blocking therapy were retrospectively identified from five Dutch centers. From these cohorts, we selected all patients who developed MAS while receiving IL‐1 pathway–targeted therapy for analysis, resulting in 22 patients with MAS receiving IL‐1–blocking therapy in total. Together, these 22 patients experienced 29 MAS episodes: 18 during anakinra treatment and 11 during canakinumab treatment (Table 1). Data on three patients who developed MAS while not receiving IL‐1–targeted therapy are available in Supplementary Table 1.

**Table 1:** Patient characteristics with SD‐associated MAS treated with (first‐line) Ana or Can*

Patient (episode)	Sex	Trisomy 21	Ethnicity	Age at diagnosis of SD, y	Arthritis at diagnosis of SD	Treatment of SD at diagnosis of MAS	Age at diagnosis of MAS, y	Duration of SD at MAS diagnosis, days	Disease status before MAS	Microbiology results	MAS episodes while using non‐IL‐1 therapy for SD	Follow‐up, y
1	F	No	Arab	6.97	No	Ana 2 mg/kg	14.16	2,625	Act	NI	No	11.53
2	M	Yes	WE	1.06	Yes	Ana 3 mg/kg, Pred 1 mg/kg	1.16	34	Act	Rotavirus	Tocilizumab	11.65
3 (1)	F	No	WE	11.64	Yes	Can, Pred <0.1 mg/kg	16.0	1,595	Act	Adenovirus	No	7.16
3 (2)						Can 1 dose per 3 wk, Pred 0.5 mg/kg	16.5	1,776	CID	NI		
3 (3)						Can 1 dose per 3 wk, Pred 0.2 mg/kg, Aza	18.69	2,578	CID	*Robinsoniella* bacteremia post ileocecal resection		
4 (1)	F	No	WE	14.7	Yes	Ana 2 mg/kg	15.33	229	CID	NI	No	7.95
4 (2)						Ana 2 mg/kg	15.92	444	CID	(primary) EBV		
5	F	No	Arab	3.18	Yes	Ana 6 mg/kg	3.18	2	Act	NI	No	6.54
6	M	Yes	WE	1.6	Yes	Ana 2 mg/kg	1.95	128	Act	Rhinovirus^a^	No	0.44^b^
7	M	No	ME	4.41	Yes	Ana 5.5 mg/kg	4.43	6	Act	NI	No	3.61
8	F	No	Arab	16.07	Yes	Ana 2 mg/kg	16.09	8	Act	NI	No	2.41
9	F	No	WE	5.03	No	Ana 2 mg/kg	5.04	2	Act	Rotavirus	No	5.28
10	M	No	WE	3.85	Yes	Can 1 dose per 8 wk	17.14	4,855	CID	(recent) EBV^a^	No	15.45
11 (1)	F	No	WE	1.62	No	Can 1 dose per 3 wk	2.48	311	CID	NI	Tofacitinib	2.92
11 (2)						Can 1 dose per 3 wk	2.82	438	Act	NI		
12	M	No	WE	3.17	No	Ana 6 mg/kg	3.56	143	CID	(primary) EBV	No	6.86
13	F	No	WE	16.61	No	Ana 4 mg/kg	16.66	18	Act	NI	No	6.22
14	F	No	ME	10.1	Yes	Ana 2 mg/kg	10.16	20	Act	NI	No	5.50
15 (1)	F	No	WE	15.72	Yes	Ana 2 mg/kg	16.39	244	Act	NI	No	5.03
15 (2)						Ana 2 mg/kg AD	17.81	763	Act	NI		
16	F	No	WE	2.08	Yes	Ana 4 mg/kg	2.15	28	Act	(recent) EBV	No	4.68
17 (1)	F	No	WE	1.48	No	Ana 2 mg/kg	1.48	1	Act	NI	No	4.68
17 (2)						Ana 4 mg/kg	2.58	402	CID	NI		
18	F	No	WE	10.96	Yes	Can 1 dose per 3 wk	11.31	130	CID	NI	No	4.24
19 (1)	M	Yes	WE	3.46	Yes	Can	5.37	698	CID	(primary) EBV^a^	No	4.26
19 (2)						Can 1 dose per 5 wk	7.15	1,348	CID	NI		
20	F	No	Asian	0.93	Yes	Ana 6.6 mg/kg	1.02	34	Act	NI	No	3.84
21	F	No	WE	11.66	Yes	Can, MTX, Pred 0.8 mg/kg	12.89	450	Act	NI	No	1.24^b^
22	F	No	WE	2.55	Yes	Can 6.3 mg/kg, MTX, Pred 0.3 mg/kg	4.53	722	Act	NI	No	4.79

* Act, active systemic juvenile idiopathic arthritis; AD, every other day; Ana, anakinra (daily dose); Aza, azathioprine (1 daily dose 100 mg); Can, canakinumab (4 mg/kg, 1 dose per 4 wk if not specified); CID, clinical inactive disease; EBV, Epstein‐Barr virus; F, female; IL, interleukin; M, male; MAS, macrophage activation syndrome; ME, multiethnic; MTX, methotrexate; NI, not identified; Pred, prednisolone; SD, Still disease; WE, Western European.

^a^ Uncertain trigger due to low levels in sample.

^b^ Follow‐up was less than 2 years due to patient death.

Among the 22 patients included (Table 1), 73% were female (16 of 22), and 14% had trisomy 21 (3 of 22). The median age at sJIA‐SD diagnosis was 4.1 years (range 0.9–16.6 years), whereas the median age at the first sJIA‐SD MAS episode was 5.2 years (range 1.0–17.1 years). Notably, 27% (6 of 22) of patients with MAS did not exhibit arthritis at sJIA‐SD diagnosis, consistent with findings from a previous sJIA‐SD cohort (29%, 12 of 42; *P* = 0.913).^8^ The majority (73%, 16 of 22) of patients were of Western European descent.

Although no specific trigger was identified in 59% (17 of 29) of MAS episodes, the most common potential trigger was a recent (primary) EBV infection (positive EBV serologic and quantitative PCR results in blood, evaluated by a pediatric microbiologist) in 17% (5 of 29) of episodes. In 38% (11 of 29) of episodes, MAS developed in patients who were in clinically inactive disease while receiving treatment. In 5 of these 11 episodes, an infectious trigger was identified (4 of 5 EBV, 1 septicemia after intestinal surgery). Additionally, 73% (16 of 22) of patients experienced their first MAS episode within the first year after diagnosis, whereas some patients developed MAS years later—up to 13 years in our cohort. The median time between the diagnosis of the first MAS episode and the sJIA‐SD diagnosis was 129 days (range 1–4,855 days). Among the 22 patients experiencing their first MAS episode under IL‐1–targeted therapy (either anakinra or canakinumab), 32% (7 of 22) also developed a second episode within the median follow‐up of 4.9 years (range 0.4–15.5 years) (Table 1). The median interval between the first and second MAS episodes was 401 days (range 127–3,646 days). In the 11 MAS episodes occurring while patients were receiving canakinumab treatment, patients were receiving a median dose of 4 mg/kg (range 4–6.3 mg/kg) every three to eight weeks. These seven patients were switched to canakinumab after a median of 409 days after sJIA‐SD diagnosis (range 19–2,051 days). MAS was diagnosed at a median of 6 days before the next dose of canakinumab (range 0–15 days). Patients were already receiving canakinumab treatment for a median of 265 days (range 41–2,146 days) before the first MAS episode.

### MAS developing during IL‐1 pathway–targeting treatment fulfills the EULAR/ACR/PRINTO 2016 criteria

In the studied cohorts, 28 of 29 MAS episodes (97%) occurring while patients were receiving IL‐1 pathway–targeted treatment (either anakinra or canakinumab) met the EULAR/ACR/PRINTO 2016 classification criteria for MAS in sJIA‐SD^26^ before the initiation of MAS‐directed therapy (Table 2). All patients developed fever, and in 28 of 29 episodes, ferritin levels exceeded the EULAR/ACR/PRINTO criteria threshold before glucocorticoid treatment initiation. Additionally, all episodes (n = 29) met at least two of the following minor criteria: platelet count ≤181 × 10^9^/L (59%, 17 of 29), AST levels >48 U/L (100%, 29 of 29), triglyceride levels >156 mg/dL (71%, 20 of 28), or fibrinogen levels ≤360 mg/dL (84%, 21 of 25). Notably, 79% of episodes met three of four minor criteria, and 21% met all four. Other clinical features included rash (55%, 16 of 29), lymphadenopathy (48%, 14 of 29), hepatomegaly (45%, 13 of 29), splenomegaly (24%, 7 of 29), and coagulopathy (28%, 8 of 29; petechiae, purpura, and/or bleeding). Central nervous system involvement was reported in one episode, manifesting as seizures. One episode featured arthritis.

**Table 2:** MAS episodes under IL‐1–targeted therapy for SD fulfill the clinical and laboratory features of the EULAR/ACR/PRINTO 2016 classification criteria at the diagnosis of MAS*

Features of EULAR/ACR/PRINTO 2016 classification criteria^26^	EULAR/ACR/PRINTO MAS 2016 classification criteria reference values	Our cohort	MAS episodes meeting the EULAR/ACR/PRINTO MAS 2016 classification criteria, % (n/N)
Clinical features, n			
Fever	Yes	100% (29/29)	100 (29/29)
Laboratory features, median (IQR)			
Ferritin, ng/mL	>684	4,458 (1,901–11,930)	97 (28/29)
And any 2 of the following minor criteria			
Platelet count, ×10^9^/L	≤181	128 (95–243)	59 (17/29)
AST, U/L	>48	188 (109–516)	100 (29/29)
Triglycerides, mg/dL	>156	201 (151–259)	71 (20/28)
Fibrinogen, mg/dL	≤360	200 (160–280)	84 (21/25)
No. of minor criteria met			
2	–	–	100 (29/29)
3	–	–	79 (23/29)
4	–	–	21 (6/29)
Overall no. of episodes meeting the 2016 classification criteria	–	–	97 (28/29) due to ferritin levels below threshold (patient 22)

* ACR, American College of Rheumatology; AST, aspartate aminotransferase; IL, interleukin; IQR, interquartile range; MAS, macrophage activation syndrome; PRINTO, Paediatric Rheumatology International Trials Organisation; SD, Still disease.

### The immunologic profile of patients developing MAS in SD differs from active sJIA‐SD

**Figure 1:**
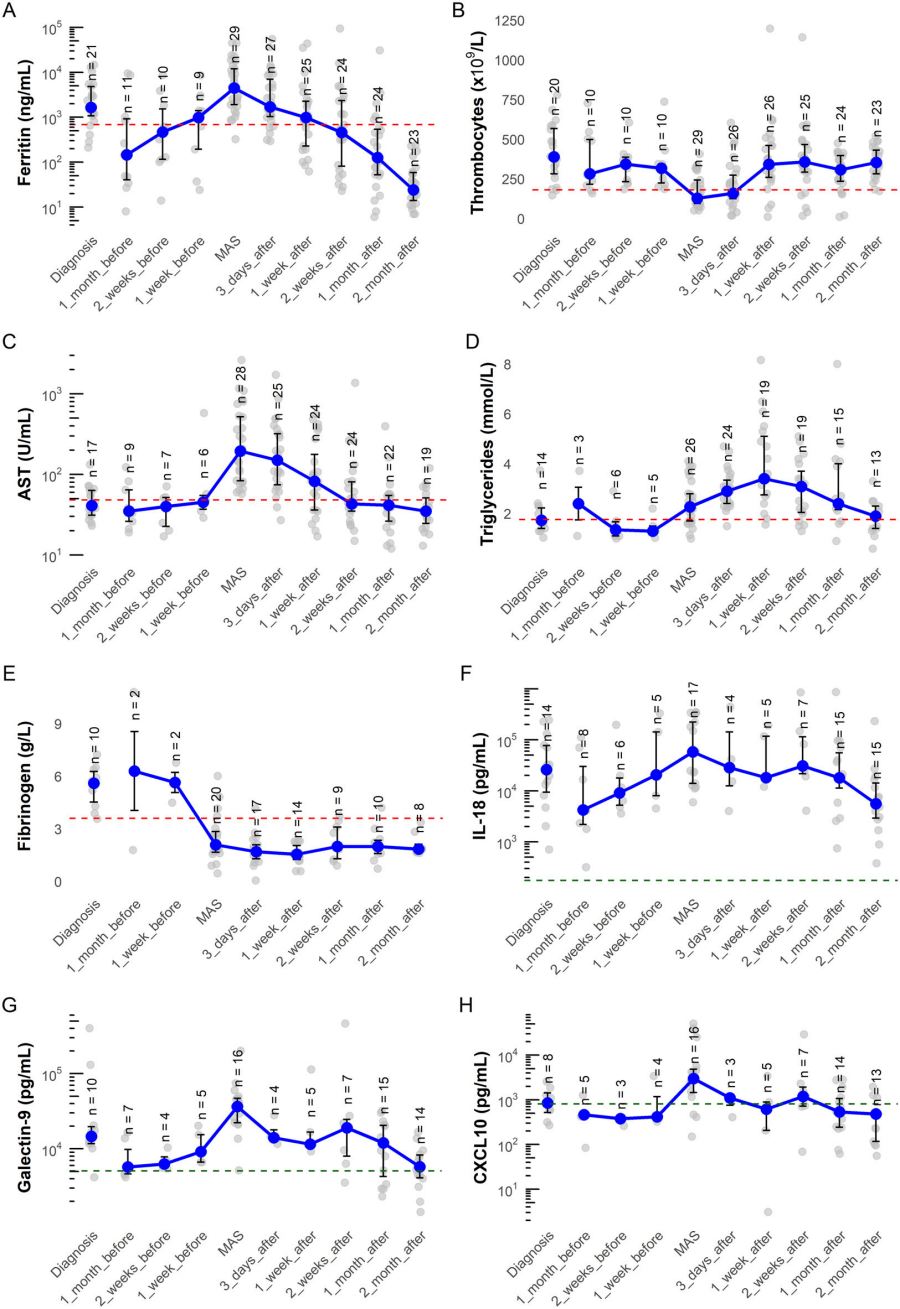
The canonical laboratory features of the EULAR/ACR/PRINTO MAS criteria and cytokine levels over time. (A) Ferritin, (B) thrombocyte, (C) AST, (D) triglyceride, (E) fibrinogen, (F) IL‐18, (G) galectin‐9, and (H) CXCL10 measurements per patient at sJIA‐SD diagnosis and per episode: one month, two weeks, and one week before MAS; MAS diagnosis (start of MAS treatment); and three days, one week, two weeks, one month, and two months after MAS therapy initiation. Blue dots indicate the median, and bars indicate interquartile range. Gray dots indicate individual measurements. The red dashed line indicates the threshold of the EULAR/ACR/PRINTO MAS criteria.^26^ The green dashed line indicates the clinical reference values. Maximum n at sJIA‐SD diagnosis is 22 patients, and maximum n at MAS diagnosis is 29 episodes. ACR, American College of Rheumatology; AST, aspartate aminotransferase; IL, interleukin; MAS, macrophage activation syndrome; PRINTO, Paediatric Rheumatology International Trials Organisation; SD, Still disease; sJIA, systemic juvenile idiopathic arthritis.

Because 11 of 22 of our patients with sJIA‐SD were observed through a protocolized prospective follow‐up schedule, we were able to sample and assess a substantial proportion of patients routinely and in close proximity (within one month) to the onset of their MAS episode. The laboratory features over time of the EULAR/ACR/PRINTO MAS criteria of our cohort are depicted in Figure 1. Lactate dehydrogenase levels and the ferritin:ESR ratio^30^ over time are depicted in Supplementary Figure 1. The MAS episodes are characterized by a marked increase in ferritin levels to very high levels and a sudden drop in platelet counts, accompanied by an increase in AST and triglyceride levels and a decrease in fibrinogen levels. This profile contrasted with the laboratory features at the diagnosis of sJIA‐SD, with elevated thrombocyte levels (median 389 × 10^9^/L [IQR 282–568× 10^9^/L] at diagnosis compared to 128 × 10^9^/L [IQR 95–243× 10^9^/L] at MAS diagnosis; *P* < 0.0001) and more moderately increased ferritin levels (median 1,655 ng/mL [IQR 1,078–4,801 ng/mL] at diagnosis compared to 4,458 [IQR 1901–11,930 ng/mL] at MAS diagnosis; *P* = 0.028). Interestingly, our data show that median ferritin levels begin to increase approximately one month in advance of a diagnosis of MAS (from 146 ng/mL to 466 and 981 ng/mL for one month, two weeks, and one week before MAS, respectively; *P* = 0.0002). In contrast, thrombocyte counts and AST, triglyceride, and fibrinogen levels remain relatively stable until MAS diagnosis.

Following the initiation of MAS therapy, ferritin levels, platelet counts, and AST levels rapidly normalized within one week to a month. Triglyceride levels, however, displayed a distinct pattern, continuing to rise even after therapy initiation before gradually normalizing. Correspondingly, fibrinogen levels, unlike other laboratory markers, remained suppressed after MAS treatment initiation and showed delayed recovery.

As expected, IL‐18 levels were elevated at the diagnosis of active sJIA‐SD in our cohort (median 26,119 pg/mL [IQR 9,400–77,118 pg/mL]) (Figure 1). Interestingly, our data reveal an increase in IL‐18 levels during the month preceding MAS, similar to ferritin levels, with IL‐18 levels peaking at diagnosis of MAS (from median 4,222 pg/mL [IQR 2,184–30,017 pg/mL] one month before diagnosis to 58,141 pg/mL [IQR 13,941–222,273 pg/mL] at MAS diagnosis; *P* = 0.011). Conversely, CXCL10 and galectin‐9 levels were more moderately increased at sJIA‐SD diagnosis and rose sharply only in the week before MAS (median galectin‐9 levels from 14,618 pg/mL [IQR 117,010–19,721 pg/mL] at diagnosis to 36,384 pg/mL [IQR 22,126–469,810 pg/mL] at MAS diagnosis [*P* = 0.041]; median CXCL10 levels from 847 pg/mL [IQR 519–1,430 pg/mL] at diagnosis to 2,986 pg/mL [IQR 1,449–4,833 pg/mL] at MAS diagnosis [*P* = 0.023]).

### First‐line anakinra treatment in new‐onset sJIA‐SD does not reduce the incidence of MAS in the first two years of disease

In the nationwide prospective ESTIS cohort, in which all patients started with first‐line anakinra, 12 of 66 (18%) developed MAS within the first two years after diagnosis. Among them, 11 patients (92%) experienced MAS while receiving IL‐1 pathway–targeted therapy (either anakinra or canakinumab), accounting for 17% of the total cohort. Additionally, 1 of 12 patients with MAS (8%) experienced a second MAS episode within the follow‐up of ESTIS, of which 1 of 13 episodes (8%) resulted in death. The percentage of patients developing MAS in the ESTIS cohort in the first two years (18%) aligns with historically reported rates, suggesting that early initiation of IL‐1–targeted monotherapy for sJIA‐SD does not reduce MAS incidence in the first two years of disease.^17^, ^20^, ^27^, ^31^, ^32^

Because all patients with new‐onset sJIA‐SD in the ESTIS cohort started anakinra at diagnosis, the rate of MAS events under anakinra treatment could be assessed in patient‐years. During the two‐year follow‐up of the prospective cohort, seven patients experienced a total of eight MAS episodes while taking anakinra. The total anakinra exposure within this period was 41.4 patient‐years, resulting in an MAS incidence of 19.3 per 100 patient‐years (95% confidence interval 8.3–38.1).

### Identification of potential demographic risk factors for MAS development

**Figure 2:**
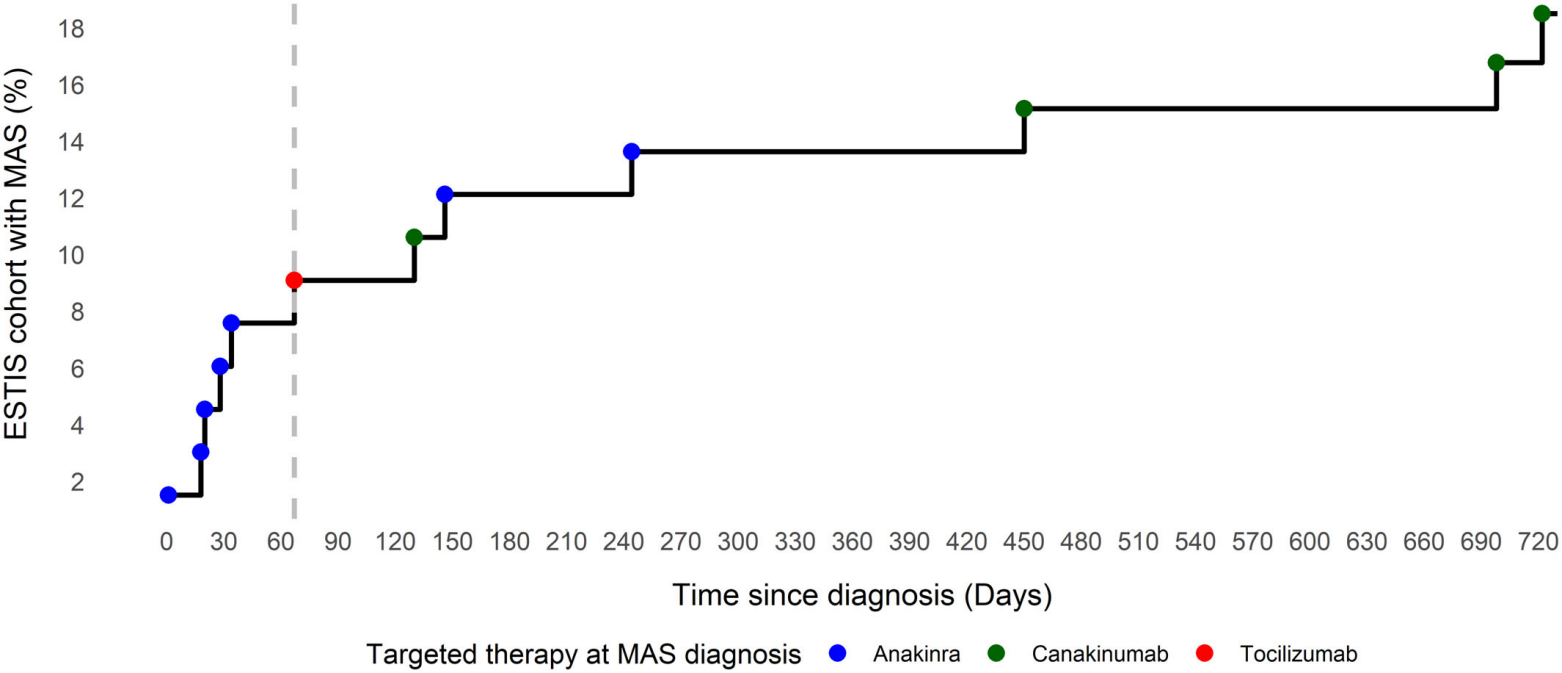
Patients are most at risk for MAS in the first three months of diagnosis. Percentage of the ESTIS cohort with MAS over time (days since diagnosis up to two‐year visit). The red dot indicates an MAS episode under IL‐6 pathway–targeted treatment (tocilizumab), blue dots indicate MAS episodes under anakinra treatment, and green dots indicate MAS episodes under canakinumab treatment. The gray dashed line indicates the time point when 50% of the first MAS episodes occurred. Total number of patients in the prospective ESTIS cohort = 66; total number of patients with MAS = 12. ESTIS, Early Stop of Targeted Treatment in Children with Systemic Juvenile Idiopathic Arthritis; IL, interleukin; MAS, macrophage activation syndrome.

Because there was a female predominance, in contrast to the typical 1:1 ratio in sJIA‐SD,^11^ and a high percentage of patients with trisomy 21 in the MAS cohort, we sought to identify if these serve as potential risk factors for developing MAS (irrespective of the type of targeted treatment used). Therefore, we compared patients with and without MAS in the ESTIS cohort. The MAS group had a higher percentage of female patients (83% [10 of 12] vs 48% [26 of 54]; *P* = 0.051) and a lower median age at sJIA‐SD diagnosis (MAS 3.32 years, range 0.93–16.6 years; no MAS 9.09 years, range 2.73–15.3 years; *P* = 0.084), but both differences did not reach statistical significance. Arthritis prevalence at sJIA‐SD diagnosis was comparable between groups (MAS 75%; no MAS 63%; *P* = 0.519). One patient with MAS had trisomy 21, compared to none in the non‐MAS group. More than half of first MAS episodes occurred within the first three months of therapy initiation (median onset 67 days; 6 of 12 episodes before three months) (Figure 2).

### MAS episode treatment characteristics and outcome

To evaluate the impact of IL‐1 pathway–targeted treatment (either anakinra or canakinumab) on MAS progression, we analyzed MAS treatment and outcomes (Table 3). Glucocorticoids were initiated in all cases, with a median prednisolone dose of 1 mg/kg (range 0.5–2 mg/kg). Intravenous methylprednisolone pulses were used in 41% (12 of 29) of episodes. Although most patients received at least part of their steroid treatment intravenously, 28% (8 of 29) of episodes were managed with oral steroids, besides the continuation of their IL‐1–targeted therapy, often in temporarily higher dosages due to MAS. The median anakinra dose increased significantly from 2 mg/kg daily (range 2–6.6 mg/kg) to 4 mg/kg daily (range 2–13 mg/kg; *P* = 0.0229). Among the patients receiving anakinra treatment, 13 of 18 episodes improved solely with the addition of steroids (including methylprednisolone pulses) and an increase of the anakinra dose. IL‐1–targeted therapy was switched to tocilizumab in 14% (4 of 29) of episodes. Cyclosporine was introduced in four episodes (14%), with the addition of dexamethasone and etoposide in one patient according to the HLH 2004 protocol.^33^

**Table 3:** Treatment and outcome of MAS episodes under IL‐1 pathway–targeted treatment for SD*

Patient (episode)	Maximum dose of steroids, mg/kg	MP pulse	Maximum Ana dose, mg/kg	Other	Maximum dose of steroids, days^a^	Treatment of MAS, days^b^	Hospitalization, days	ICU admission	Outcome
1	1	No	4	–	8	127	0	No	Recovery MAS, continued maintenance therapy SD (switch to Can)
2	1	No	8	CSA, Eto, Dexa	14	60	92	No	Recovery MAS, continued maintenance therapy SD until next MAS (Toci)
3 (1)	0.5	No	–	Can frequency increased to 1 dose per 3 wk	12	–	0	No	Continued maintenance therapy until next MAS
3 (2)	1	Yes	–	–	7	117	9	No	Recovery MAS, continued maintenance therapy SD until next MAS
3 (3)	2	Yes	4.5	CSA, Ana IV, emapalumab	No taper or stop	–	37	Yes	Initial improvement after MP pulse, 5 days later return of fever, start CSA and MP pulse and Ana IV; ICU hemodynamic instability, start emapalumab, initial improvement but return of fever; rapid progression, multiorgan failure due to circulatory shock, death
4 (1)	1	No	2	–	13	105	2	No	Recovery MAS, maintenance therapy SD until next MAS
4 (2)	1	Yes	4	–	14	231	9	No	Recovery MAS, continued maintenance therapy SD (switch to Can, 5 y later stop)
5	1	Yes	6	–	7	35	21	No	Recovery MAS, 6 mo later stop Ana; 1 y later restart and switch to Can; eventually 1 y later stop
6	1	Yes	10	CSA, switch to Toci, Ana IV	No taper or stop	–	56	Yes	MAS with quick respiratory and circulatory deterioration, intubation, MP pulses, CSA and Toci; no response; start Ana IV with initial response and return to SC; development of ARDS restart Ana IV, MP pulse, and Toci without response; multiorgan failure, death
7	1	Yes	6	–	15	63	18	No	Recovery MAS, 5 mo later stop Ana, 3 y later flare and start Can after 3 mo stop
8	1	No	3	–	7	20	3	No	Recovery MAS, recovery SD
9	1	No	4	Dexa, switch to Toci, switch back to Ana	7	137	47	No	Recovery MAS, recovery SD
10	0.5	No	–	Can frequency increased to 1 dose per 4 wk	7	70	4	No	Recovery MAS, continued maintenance therapy SD
11 (1)	1	Yes	–	Can dose increased to 6 mg/kg	31	106	3	No	Recovery MAS, continued maintenance therapy SD until next MAS
11 (2)	1	No	–	Switch to Toci	7	–	0	No	Continued maintenance therapy until next MAS (tofacitinib)
12	1	No	6	–	7	153	6	No	Recovery MAS, recovery SD
13	1.2	No	4	–	14	164	0	No	Recovery MAS, continued maintenance therapy SD; stop 3.5 y later, restart of therapy after 2 mo, eventually stopping treatment 1 y later
14	1.5	No	2	–	6	Unable to stop >2 y	4	No	Recovery MAS, month later switch to Can, 6 mo later switch to Toci; 4.5 y later stop therapy
15 (1)	0.5	No	2	–	8	133	0	No	Recovery MAS, continued maintenance therapy SD until next MAS
15 (2)	0.6	No	2	Ana from AD to daily	16	273	0	No	Recovery MAS, continued maintenance therapy SD (switch to Can)
16	1.2	No	4	–	9	97	3	No	Recovery MAS, recovery SD
17 (1)	2	Yes	4.5	CSA	11	168	31	No	Recovery MAS, continued maintenance therapy SD until next MAS
17 (2)	1.25	Yes	8	–	10	–	3	No	Unable to discontinue glucocorticoids, switch to Can; clinical improvement but continued smoldering liver isolated MAS (confirmed with liver biopsy); received 1 mg/kg prednisolone, MP pulse therapy and CSA, resulting in clinical improvement and normalization of liver values; subsequently developed sJIA‐LD (confirmed with HRCT); with addition of tofacitinib to Can stable clinic
18	1	No	–	Can frequency increase to 1 dose per 3 wk	3	203	3	No	Recovery MAS, continued maintenance therapy SD
19 (1)	0.5	No	–	–	34	73	0	No	Recovery MAS, continued maintenance therapy SD until next MAS
19 (2)	1.5	No	–	Can dose increased to 5 mg/kg	4	81	5	No	Recovery MAS, continued maintenance therapy SD (6 mo after restart prednisolone 0.5 mg/kg)
20	1	Yes	13	–	14	67	4	No	Recovery MAS, Recovery SD
21	1	Yes	4	IVIG, Ana IV	No taper or stop	–	9	Yes	Possible sJIA‐LD‐like pulmonary image; ICU hemodynamic instability, very rapid progression, cerebral herniation, death
22	1	Yes	–	Stop MTX, switch to Toci	28	91	5	No	Recovery MAS, continued maintenance therapy SD

* Recovery MAS was defined as patients who have safely stopped their medication for MAS; recovery SD was defined as patients who have safely stopped their medication for SD within 1 y after MAS without restart of therapy up until the last follow‐up. AD, every other day; Ana, anakinra; ARDS, acute respiratory distress syndrome; Can, canakinumab; CSA, cyclosporine A; Dexa, dexamethasone; Eto, etoposide; HRCT, high‐resolution computed tomography; ICU, intensive care unit; IL, interleukin; sJIA‐LD, sJIA associated lung disease; IV, intravenous; IVIG, intravenous immunoglobulin; MAS, macrophage activation syndrome; MP, methylprednisolone; MTX, methotrexate; SC, subcutaneous; SD, Still disease; Toci, tocilizumab.

^a^ Time (in days) that patients received the maximum dose of glucocorticoids (before tapering).

^b^ The time (in days) that patients received steroids as a treatment regimen for MAS.

Patients began tapering steroids after a median of 10 days (range 3–34 days) and discontinued glucocorticoids after a median of 106 days (range 20–273 days) (Table 3). Hospitalization occurred in 76% (22 of 29) of episodes, with a median stay of 4 days (range: 2–92 days). Intensive care unit (ICU) admission was required in 10% (3 of 29) of cases, and 14% of patients (3 of 29 cases [10%]) died due to uncontrolled sJIA‐SD–associated MAS despite combined immunosuppressive treatments. Overall, 17% (5 of 29) of cases (23% of patients) achieved recovery of MAS with successful tapering and discontinuation of sJIA‐SD therapy within a year, and 72% (21 of 29) of cases achieved complete recovery of MAS but required ongoing sJIA‐SD maintenance therapy for at least one year after MAS, or a second episode developed before therapy could be discontinued. One patient (patient 17 in Table 3) with refractory sJIA‐SD and recurrent MAS eventually developed sJIA‐LD (digital clubbing and septal thickening with ground glass opacities on high‐resolution computed tomography).^13^ Bone marrow aspiration was performed in two patients, revealing hemophagocytosis in one.

## DISCUSSION

To explore the development and occurrence of MAS and the applicability of the 2016 MAS criteria in patients with sJIA‐SD receiving IL‐1–targeted therapy (either anakinra or canakinumab), we analyzed clinical and laboratory characteristics from six Dutch pediatric rheumatology centers where anakinra is used as first‐line therapy in the national treatment guideline. We found that the EULAR/ACR/PRINTO 2016 classification criteria^26^ proved to be accurate and applicable to patients with sJIA‐SD who develop MAS while receiving IL‐1 pathway–blocking therapy. Furthermore, we showed, using data from a nationwide prospective cohort study, that although early initiation of IL‐1–blocking therapy has improved the outcome for SD in general, the incidence of MAS in patients with sJIA‐SD treated with first‐line anakinra in the first two years post diagnosis does not seem to be meaningfully changed when compared to historic cohorts.^17^, ^20^, ^27^, ^31^, ^32^ Primary EBV infection was the most common potential infectious trigger identified in our cohort. Patients seem to be particularly vulnerable to MAS development during the early disease stages of sJIA‐SD, as most MAS episodes in our cohort occurred in the first three months after diagnosis. One‐third of patients experienced recurrent MAS episodes. Finally, our data suggest that female sex, disease onset at a young age,^34^ and trisomy 21 are potential risk factors for MAS development. These and other potential risk factors for MAS that we have not identified or studied but that have been described in the literature, such as atypical (pruritic) rashes,^35^ warrant further research in larger cohorts.

Because active SD and disease flares are well‐known triggers of MAS episodes^15^, ^17^ and IL‐1 blockade has demonstrated high efficacy in inhibiting disease activity and improving SD outcome,^8^, ^11^, ^12^, ^24^ a reduction in MAS incidence might have been expected in our multicenter cohort with anakinra as first‐line treatment. However, despite early initiation of anakinra, we observed an incidence of MAS of 18% in the first two years after diagnosis (in 11 of 12 patients while still using IL‐1–targeted therapy), which is in line with (and not lower than) the percentages reported in patients treated with glucocorticoids and/or DMARDs for sJIA‐SD.^17^, ^20^, ^27^, ^31^, ^32^ This is, to the best of our knowledge, the first time that the incidence of sJIA‐SD–associated MAS is estimated from a national multicenter inception cohort consisting of patients who were treated with first‐line anakinra monotherapy in a prospective study treatment protocol.

Because early start of IL‐1 pathway blockade alone does not appear to reduce MAS incidence, additional immune pathways likely contribute to MAS pathogenesis. Unlike active SD, MAS is characterized by elevated IFNγ levels, which plays a central role in MAS development and perpetuation, as demonstrated by the efficacy of emapalumab, an anti‐IFNγ monoclonal antibody, in treating MAS.^16^, ^36^, ^37^ Recently, SD‐associated MAS has also been shown to be characterized by type 1 IFN pathway activation.^38^ Moreover, IL‐18, which is elevated in almost all cases of active SD, reaches extreme levels in MAS. Persistently high IL‐18 levels despite maintenance treatment have been associated with an increased MAS risk, highlighting its potential pathogenic role and therapeutic relevance for daily clinical practice.^39^, ^40^, ^41^, ^42^ Indeed, the different temporal dynamics of IL‐18, ferritin, CXCL10, and galectin‐9 observed in our cohort might suggest IL‐18 and ferritin as early indicators of escalating inflammation and risk for developing MAS, whereas the later increase in CXCL10 and galectin‐9 levels, both reflecting the activation of IFN signaling,^16^, ^43^, ^44^, ^45^ may be interpreted as the transition to overt MAS. These findings underscore the need for increased vigilance in patients with persistently high or rapidly increasing IL‐18 levels and the value of monitoring IFN‐induced chemokines, such as CXCL9 and CXCL10 (IFNγ) or galectin‐9 (type 1 IFN), because these patterns may signal impending MAS.^16^, ^22^, ^39^, ^40^

We identified EBV infections in 17% of the MAS episodes as the most likely trigger (by positive EBV serologic and quantitative PCR testing results). This is interesting because EBV infection has been reported to uniquely trigger high levels of IL‐18, IFNγ‐induced chemokines, and vastly expanded CD38^+^HLA–DR^+^ T lymphocytes,^46^ a subset of (pathogenic) T cells that has been associated with IFNγ production in MAS.^38^

Thanks to the characteristics and inclusion criteria of our prospective cohort, we were able to evaluate the incidence of MAS specifically for patients receiving anakinra treatment, which is 19.3 per 100 patient‐years. This is significantly higher than that in the patients with sJIA‐SD taking canakinumab reported by Grom et al (2.8 per 100 patient‐years, *P* = 0.0001).^18^ However, the latter cohort consisted of patients with active sJIA‐SD with established disease, a median of more than two years post diagnosis, and a higher likelihood of steroid exposure. In contrast, our cohort consisted of patients with new‐onset sJIA who started with anakinra monotherapy, of whom ~70% continued to take anakinra monotherapy and of whom >50% were able to successfully taper and stop treatment within 12 months after diagnosis.^6^, ^11^ The differences in disease phase and cotreatment with glucocorticoids next to IL‐1–targeted therapy likely contribute to the observed difference in MAS incidence.

A direct comparison of MAS incidence between anakinra‐ and canakinumab‐treated patients within our cohort is liable to multiple confounding factors. Notably, only a specific subset of patients—those who do not respond adequately to anakinra or who are unable to taper—are subsequently treated with canakinumab or tocilizumab, whereas all patients initially receive anakinra. Because MAS risk appears to be highest early in the disease course,^17^, ^47^ as supported by our findings (50% of episodes occurred within the three months after diagnosis), and all patients begin treatment with anakinra, they are inherently more likely to develop MAS while still taking anakinra.

The median time from sJIA‐SD diagnosis to MAS diagnosis in the ESTIS cohort was 64 days, markedly earlier than the 107‐day median reported in glucocorticoid‐treated patients.^17^ This, together with the previously described results, suggests that early and targeted treatment using anakinra as a first‐line strategy, with its narrower immunomodulatory profile compared to glucocorticoids, may not be sufficient in suppressing the different mechanisms underlying MAS development in some patients with sJIA‐SD, particularly in the first months of disease.^48^ Although we reported a high success rate of first‐line use of anakinra on SD outcome in general, with markedly reduced necessity for glucocorticoid use in the first two years of disease,^11^ it may be beneficial to consider temporary (eg, four to six weeks) use of (low‐dose) glucocorticoids adjacent to anakinra in the very early phase of SD. Another explanation for the relatively high incidence of MAS in the first months after sJIA‐SD diagnosis may be that the on‐label dose of anakinra at sJIA‐SD diagnosis (2 mg/kg) is too low to prevent MAS development in some patients. Further research is needed to confirm these observations. In addition, our data warrant close monitoring of EBV‐seronegative patients with SD and of patients with persistent inflammation (high or increasing ferritin and/or IL‐18 levels) and therefore an increased risk for MAS development.

Compared to the cohort in the study by Minoia et al,^17^ which described 362 MAS cases, our cohort showed a significantly lower frequency of cyclosporine administration (61% vs 14%; *P* < 0.0001) and ICU admissions (35% vs 10%; *P* = 0.0076). Nonetheless, mortality rates remained similar (8% vs 10%; *P* = 0.669). Further research is needed to confirm these observations and determine the optimal management approach in this setting.

Importantly, 28 of 29 MAS episodes in our cohort of patients treated with IL‐1 blockade met the EULAR/ACR/PRINTO 2016 criteria, supporting their applicability in this clinical setting. Interestingly, we did observe ~50% lower median ferritin levels in our patients when compared to historical glucocorticoid‐treated cohorts (Supplementary Table 2).^17^, ^26^, ^28^ Also, the single MAS episode that did not fulfill the criteria displayed a ferritin level below the threshold at the time of MAS diagnosis. This aligns with previous findings by Schulert et al,^27^ who reported lower ferritin levels in canakinumab‐treated patients compared to historical controls, and Ulu et al,^47^ who observed reduced ferritin levels in patients with MAS treated with anakinra and canakinumab. Additionally, in 41% of MAS episodes in our cohort, platelet counts remained above the threshold of the classification criteria. This might be, next to a direct effect of IL‐1 pathway inhibition on the biochemical profile of MAS,^15^ at least partially due to early diagnosis and initiation of MAS treatment in our cohort. This is supported by findings from a Japanese cohort, in which patients in the early phase of MAS had lower ferritin levels and met the platelet count criterion in only 18% of cases, compared to 80% in full‐blown MAS.^49^ Overall, these criteria serve only to aid in the diagnosis of MAS, as changes in individual patient characteristics that raise the suspicion of MAS development are far more important than the rise or fall of laboratory values above or below a specific threshold.

There are some limitations of this study to consider. First, because we investigate a very rare disease, this study describes data from 22 patients with a total of 29 episodes of MAS, making up a relatively small cohort. However, this is the first multicenter cohort study describing pediatric patients with SD‐associated MAS while being treated uniformly in a study protocol using first‐line anakinra, minimizing the use of systemically administered glucocorticoids. Replication of our findings in other, preferably larger, cohorts is warranted. It is also important to note that in the ESTIS cohort, patients with an MAS episode coinciding with the diagnosis (therefore before the start of anakinra treatment) were excluded. Considering that, the amount of episodes early in the disease course calculated from the ESTIS cohort is most likely an underestimation. Furthermore, 20 of 22 patients with MAS were diagnosed after 2016, whereas the EULAR/ACR/PRINTO classification criteria^26^ were already published and implemented in clinical practice. Analyzing whether the patients could be diagnosed based on the EULAR/ACR/PRINTO 2016 classification criteria is therefore liable to bias. However, within the Utrecht and ESTIS cohorts, no other potential episodes of MAS for which glucocorticoid treatment was initiated were identified.

In conclusion, although first‐line anakinra has been shown to achieve high response rates and minimize glucocorticoid use and thus glucocorticoid‐related side effects in sJIA‐SD in general,^6^, ^9^, ^11^ our study suggests that early initiation of IL‐1 pathway–blocking therapy does not decrease the incidence of MAS. Importantly, the EULAR/ACR/PRINTO 2016 classification criteria^26^ remain accurate and applicable to aid in the diagnosis of MAS in patients with sJIA‐SD receiving IL‐1 pathway–targeted treatment (either anakinra or canakinumab). Our findings support close monitoring of clinical and laboratory markers in the first three months after sJIA‐SD onset in all patients, particularly female patients, those with trisomy 21, EBV‐naive patients, and those with onset of sJIA‐SD at a young age. Our findings highlight the need for further studies to identify and validate risk factors and biomarkers and optimize management strategies for this complex patient population.

## AUTHOR CONTRIBUTIONS

All authors contributed to at least one of the following manuscript preparation roles: conceptualization AND/OR methodology, software, investigation, formal analysis, data curation, visualization, and validation AND drafting or reviewing/editing the final draft. As corresponding author, Dr Vastert confirms that all authors have provided the final approval of the version to be published and takes responsibility for the affirmations regarding article submission (eg, not under consideration by another journal), the integrity of the data presented, and the statements regarding compliance with institutional review board/Declaration of Helsinki requirements.

## ROLE OF THE STUDY SPONSOR

Sobi had no role in the study design or in the collection, analysis, or interpretation of the data, the writing of the manuscript, or the decision to submit the manuscript for publication. Publication of this article was not contingent upon approval by Sobi.

## Supporting information


**Disclosure form**.

**Supplementary figure 1:** Laboratory features of Macrophage Activation Syndrome (MAS) over time Ferritin : Erythrocyte sedimentation rate (ESR) ratio (A), Lactate dehydrogenase (B) measurements per patient at sJIA‐SD diagnosis and per episode: 1 month, 2 weeks and 1 week before MAS, MAS diagnosis (start of MAS treatment), 3 days, 1 week, 2 weeks, 1 month and 2 months after MAS therapy initiation. Blue dots indicate the median with bars indicating interquartile range. Gray dots indicate individual measurements. Purple dashed line indicates the threshold for MAS of the ferritin : ESR ratio set by Eloseily et al. 2019 [PMID: 31777812]. Green dashed line indicates the clinical reference value. Maximum n at sJIA‐SD diagnosis is 22 patients and at MAS diagnosis is 29 episodes.

**Supplementary table 1:** Patient characteristics, Treatment and outcome of Macrophage Activation Syndrome (MAS) episodes without Interleukin (IL)‐1 pathway targeted maintenance treatment for Stills disease (SD)
**Supplementary table 2:** Comparison of cardinal clinical and laboratory features of the EULAR/PRINTO/ACR 2016 MAS classification criteria
