# Supplementary figures and images for: Impact of Interleukin‐1 Blockade on the Development of Macrophage Activation Syndrome in Still Disease: Incidence and Diagnostic Validity of the EULAR/ACR/PRINTO 2016 MAS Classification Criteria
Source: Arthritis Rheumatol. 2025 Aug 9;77(12):1784–98. doi: 10.1002/art.43263 (PMC12750127; doi:10.1002/art.43263)

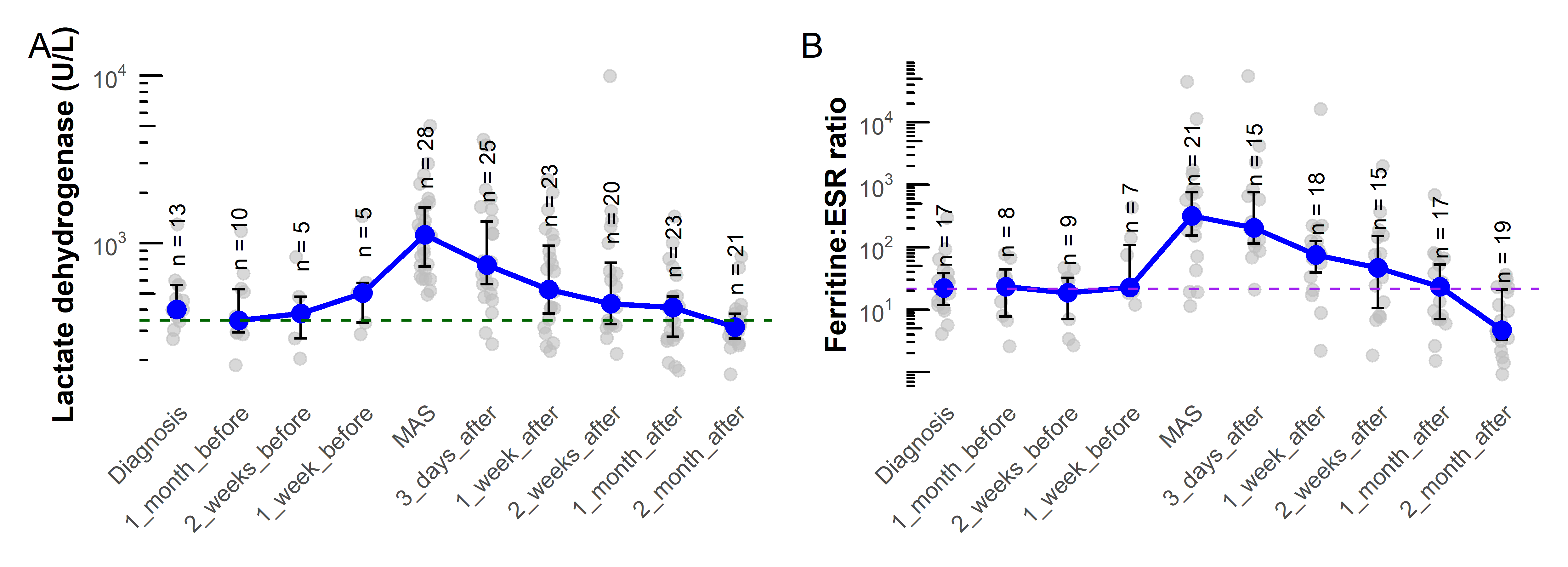

Supplement: Supplementary file 2 — Supplementary figure 1: Laboratory features of Macrophage Activation Syndrome (MAS) over time Ferritin : Erythrocyte sedimentation rate (ESR) ratio (A), Lactate dehydrogenase (B) measurements per patient at sJIA‐SD diagnosis and per episode: 1 month, 2 weeks and 1 week before MAS, MAS diagnosis (start of MAS treatment), 3 days, 1 week, 2 weeks, 1 month and 2 months after MAS therapy initiation. Blue dots indicate the median with bars indicating interquartile range. Gray dots indicate individual measurements. Purple dashed line indicates the threshold for MAS of the ferritin : ESR ratio set by Eloseily et al. 2019 [PMID: 31777812]. Green dashed line indicates the clinical reference value. Maximum n at sJIA‐SD diagnosis is 22 patients and at MAS diagnosis is 29 episodes. [file ART-77-1784-s002.tif]
