# Supplementary material for: Impact of Interleukin‐1 Blockade on the Development of Macrophage Activation Syndrome in Still Disease: Incidence and Diagnostic Validity of the EULAR/ACR/PRINTO 2016 MAS Classification Criteria
Source: Arthritis Rheumatol. 2025 Aug 9;77(12):1784–98. doi: 10.1002/art.43263 (PMC12750127; doi:10.1002/art.43263)
Supplement: Supplementary file 3 — Supplementary table 1: Patient characteristics, Treatment and outcome of Macrophage Activation Syndrome (MAS) episodes without Interleukin (IL)‐1 pathway targeted maintenance treatment for Stills disease (SD) Supplementary table 2: Comparison of cardinal clinical and laboratory features of the EULAR/PRINTO/ACR 2016 MAS classification criteria [file ART-77-1784-s003.docx]

**Supplementary table 1:** Patient characteristics, Treatment and outcome of Macrophage Activation Syndrome (MAS) episodes without Interleukin (IL)-1 pathway targeted maintenance treatment for Stills disease (SD)

| **Patient, (episode)** | **Sex** | **Trisomy 21** | **Ethnicity** | **Age diagnosis SD, years** | **Treatment SD at diagnosis MAS** | **Age diagnosis MAS, years** | **Duration SD at MAS diagnosis, days** | **Disease status before MAS** | **Possible trigger** |
| --- | --- | --- | --- | --- | --- | --- | --- | --- | --- |
| 23 | M | No | WE | 8.30 | Toci 1/2w + pred 0.1 mg/kg/d | 8.93 | 227 | CID | (Recent) EBV |
| 24 (1) | F | No | WE | 16.55 | None | 16.55 | 0 | Act | NI |
| 24 (2) |  |  | WE |  | Toci 1/w + pred 0.6 mg/kg/d + aza | 17.47 | 335 | Act | Entero/rhinovirus |
| 25 | F | No | WE | 1.68 | Toci 1/2w + pred 0.5 mg/kg/d | 1.86 | 67 | Act | Enterovirus |
|  |  |  |  |  |  |  |  |  |  |
| **Patient (episode)** | **Max dose steroids mg/kg** | **MP pulse** | **Treatment** | **Max dose steroids, days** | **Treatment MAS, days** | **Hopitalisation, days** | **ICU admission** | **Outcome** | **Follow-up, years** |
| 23 | 1 | No |  | 9 | 179 | 4 | No | Recovery MAS, continued maintenance therapy SD. | 8.67 |
| 24 (1) | 1 | Yes | Ana 2mg/kg | 10 | 31 | 6 | No | Recovery MAS, continued maintenance therapy until next MAS. 3 months later switch to Can and pred. 1 month later pred stop. 5 months later restart pred because of transaminitis. 1 months later addition of Aza and switch Can to Toci. | 2.10 |
| 24 (2) | 1 | Yes | Ana 4mg/kg, stop toci and AZA, Start CSA, emapalumab | 15 | >1 year | 25 | No | Recovery MAS, continued maintenance therapy. Initial improvement after MP pulse, switch to Ana and start CSA. Week later return of fever, start MP pulse and emapalumab. 5 months later Ana 2mg/kg/d. 2 months later start Tofa. 1 months later stop CSA. 4 months later Ana AD. |  |
| 25 | 2 | Yes | CSA | 10 | 132 | 13 | No | Recovery MAS, continued maintenance therapy. 2 months later switch to Can. 8 months later start MTX. | 2.34 |
| *MAS = Macrophage Activation Syndrome; F = Female; M = Male; WE = Western-European; Ana = anakinra (daily dose); Pred = prednisolone; MP = Methylprednisolone; CSA = Cyclosporine A; Toci = tocilizumab; Aza = azathioprine; MTX; methotrexate; Tofa = tofacitinib; ACT = Active sJIA; CID = Clinical Inactive Disease; EBV= Epstein Barr Virus; NI= Not Identified; Max dose steroids, days= time (in days) that patients received the maximum dose of corticosteroids (before tapering); Treatment MAS, days= the time (in days) that patients received steroids as a treatment regime for MAS; Recovery MAS was defined as patients who have safely stopped their medication for MAS; recovery SD was defined as patients who have safely stopped their medication for SD within one year after MAS without restart of therapy up until the last follow-up.* | | | | | | | | | |

**Supplementary table 2:** Comparison of cardinal clinical and laboratory features of the EULAR/PRINTO/ACR 2016 MAS classification criteria

| **MAS characteristics** | **Our cohort** | **Ravelli *et al.* 2016^26^** | **Minoia *et al.* 2014^17^** | **Shimizu *et al.* 2020^28^** |
| --- | --- | --- | --- | --- |
| **Clinical features, percentage (n)** | | | | |
| Fever | 100% (29/29) | 99% (93/94) | 96% (341/355) | 94% (17/18) |
| Arthritis | 3% (1/29) |  | 65% (230/354) |  |
| Rash | 55% (16/29) |  |  |  |
| Of which pruritic | 31% (5/16) |  |  |  |
| Lymphadenopathy | 48% (14/29) | 53% (48/91) | 51% (178/346) | 33% (6/18) |
| Hepatomegaly | 45% (13/29) | 72% (68/94) | 70% (245/350) | 39% (7/18) |
| Splenomegaly | 24.1% (7/27) | 58% (53/92) | 58% (201/347) |  |
| CNS dysfunction | 3% (1/29) | 43% (40/93) | 35% (122/349) | 0% (0/18) |
| Coagulopathy (petechiae, purpura and/or bleeding) | 28% (8/29) | 27% (25/92) | 20% (71/348) |  |
| **Laboratory characteristics, median (IQR)** | | | | |
| Ferritin, ng/mL | 4458 (1901-11930) | 9094 (2000-19767) | 8302 (1786-21975) | 9235 (5559-18710) |
| Platelet count, x10^9^/liter | 128 (95-243) | 98 (57-141) | 144 (86-270) | 189 (133–218) |
| AST, units/Liter | 188 (109-516) | 171 (98–436) | 116 (48-281) | 89 (61-425) |
| Triglycerides, mg/dL | 201 (151-259) | 267 (192–358) | 244 (165-356) | 214 (143-285) |
| Fibrinogen, mg/dL | 200 (160-280) | 220 (148–345) | 220 (140-370) | 294 (240-362) |
| Percentage with number of episodes; AST = AST= aspartate aminotransferase; median with interquartile range (IQR). | | | |  |
